# Outcomes of Post‐Transplant Rh‐GCSF and Decitabine Maintenance Therapy in Patients With High‐Risk Myeloid Neoplasm

**DOI:** 10.1002/ajh.70018

**Published:** 2025-07-29

**Authors:** Mathias Palmer, Anmol Baranwal, Rami Basmaci, Khalil Hassan, Jade Kutzke, Gabriel Bartoo, Hemant Murthy, James Foran, Abhishek A. Mangaonkar, Mehrdad Hefazi, Aasiya Matin, William J. Hogan, Mark Litzow, Mithun Vinod Shah, Hassan B. Alkhateeb

**Affiliations:** ^1^ Division of Internal Medicine, Mayo Clinic Rochester Minnesota USA; ^2^ Division of Hematology, Mayo Clinic Rochester Minnesota USA; ^3^ Cancer Centers of Southwest Oklahoma Lawton Oklahoma USA; ^4^ Division of Pharmacy, Mayo Clinic Rochester Minnesota USA; ^5^ Division of Hematology, Mayo Clinic Jacksonville Florida USA

**Keywords:** neoplasia‐myeloid leukemia and dysplasia, stem cell transplantation, stem cell transplantation clinical results in acute leukemia

## Abstract

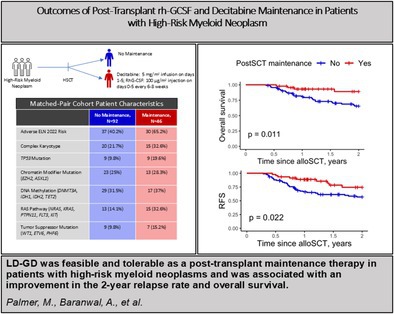

To the Editor,

Allogeneic hematopoietic stem cell transplantation (allo‐HSCT) remains the only potentially curative treatment for patients with high‐risk myeloid neoplasms (HR‐MN) [1]. Despite the recent progress in donor selection, graft versus host disease (GVHD) prophylaxis, and supportive care, disease relapse remains a major cause of death post‐transplant.

Currently, several prospective ongoing clinical trials (i.e., AMADEUS, VIALE‐T) are assessing the role of maintenance therapy following HSCT. In 2020, Gao et al. evaluated the role of low‐dose granulocyte colony‐stimulating factor (G‐CSF) with decitabine (LD‐GD) as a post‐transplant maintenance strategy. This demonstrated a significant improvement in the cumulative incidence of 2‐year relapse (HR 0.32, *p* < 0.01) [2]. However, the trial was mainly restricted to young patients with a median age of 30 years, who underwent allo‐HSCT for only high‐risk AML following myeloablative conditioning (MAC) therapy, and who achieved minimal residual disease (MRD) negative disease prior to initiating maintenance therapy. Thus, the applicability of this approach to an older population with HR‐MN following reduced‐intensity conditioning is unknown.

Since 2020, we adapted LD‐GD for patients with HR‐MN who underwent allo‐HSCT, irrespective of patient age, conditioning intensity, disease type, or MRD status prior to transplant. In this study, we describe our experience of LD‐GD and compare outcomes to a propensity‐score matched control cohort.

This study was approved by the Mayo Clinic Institutional Review Board. Please see the supplement for the detailed methods.

A total of 52 patients with HR‐MN who received LD‐GD maintenance were identified (26 males, 50%); 2 patients received LD‐GD after morphological relapse and were excluded from the analysis. Median follow‐up after transplant was 24.6 months (95% CI: 15.7–30.6 months).

Of the 50 evaluable patients in the LD‐GD cohort, 40 (80%) had AML, eight had MDS (16%), and one (2%) each had MPAL and CMML. The median age at transplant was 65 years (range 26–76 years). All MDS patients had high/very high‐risk disease; among patients with AML, 31 (78%) had adverse risk disease (Table S1). Thirty‐six patients (72%) had a matched unrelated donor, 11 (22%) had a matched related donor, and three (6%) had a haploidentical donor. Most patients received reduced intensity conditioning (*n* = 40, 80%). Patients received a median of 4 (R: 1–6) LD‐GD cycles; 14 patients (28%) completed 6 cycles. All patients were in CR, CRi, or marrow CR prior to initiating LD‐GD. The median OS and RFS were not reached (Table S2, Figure S1).

The estimated 1‐year OS and RFS were 87.3% (95% CI: 78.2%–97.4%) and 83.5% (95% CI: 73.7%–94.7%), respectively. The estimated 2‐year OS and RFS were 83.6% (95% CI: 72.9%–96.0%) and 70.5% (95% CI: 57.3%–86.9%), respectively. Subgroup analysis by cytogenetic and molecular subgroups can be found in the supplemental information (Tables S2, S3). The cumulative risk of relapse was 8.0% and 21.0% at 1 and 2 years, respectively (Figure S2). Relapse occurred in eight (16%) patients with a median time of 10.4 months (IQR: 4.8–17.1) from transplant. Among relapsed patients, five had AML and three had MDS with > 10% blasts. All relapsed patients had either complex karyotype (CK), monosomal karyotype (MK), or both complex and monosomal karyotype (CK + MK) cytogenetic profiles. Twenty‐five patients (50%) had grade 3–4 adverse events; 22 (88%) related to cytopenia and seven (28%) related to infection. Following maintenance, grade III‐IV acute graft versus host disease (aGVHD) occurred in five (10%) patients and steroid‐requiring chronic GVHD (cGVHD) occurred in 11 patients (22%), with severe cGVHD occurring in three patients (6%). A total of 10 (20%) deaths occurred at the time of last follow‐up, four related to disease progression, while six were attributed to nonrelapse mortality (4 sepsis, 1 stroke, 1 hemorrhage).

A total of 219 patients were analyzed for propensity‐score matching (173 control, 46 LD‐GD); four LD‐GD patients were excluded due to relapse before day +100 or diagnosis of CMML/MPAL (Table S6). The median age at allo‐HSCT was 64 years (IQR: 56–67 years) and the median follow‐up after transplant was 33.6 months (95% CI 28.8–37.2). The median time to maintenance initiation was 109 days after transplant (IQR: 87–147 days).

A total of 138 patients were included after propensity score matching (Table 1, Figures S3, S4). Patients receiving maintenance had a significantly lower relapse rate at 1 year after transplant (4.3% vs. 23.5%, *p =* 0.007). At 2 years post‐transplant, relapse rates favored maintenance LD‐GD (18.7% vs. 26.2%, *p =* 0.11) (Figure S5). The 2‐year RFS was superior in patients who received maintenance therapy (74.5% vs. 56.9%, *p =* 0.022) (Figure 1a, S6a) and a strong trend towards better RFS was also observed after evaluating maintenance therapy as a time‐dependent covariate (73.4% vs. 58.2%, *p =* 0.069) (Figure S6b). The 2‐year OS was also higher in patients receiving maintenance therapy (89.0% vs. 65.6%, *p =* 0.01, Figures 1b, S7a). Evaluating post‐transplant maintenance therapy as a time‐dependent covariate showed that LD‐GD was associated with an improved 2‐year OS (2‐year OS rate 88.7% vs. 66.2%, *p =* 0.026, Figure S7b). Median OS of the entire cohort was not reached.

**TABLE 1:** Patient characteristics of LD‐GD cohort and propensity‐score matched control cohort.

Variable	Control (*N* = 92)	LD‐GD (*N* = 46)	*p*
Median age at diagnosis [Min, Max]	61.0 [18.9, 75.2]	65.5 [26.2, 75.9]	0.01
Median age at treatment [Min, Max]	61.4 [19.1, 76.2]	65.9 [26.6, 76.5]	0.01
Sex (Female)	36 (39.1%)	22 (47.8%)	0.43
Acute myeloid leukemia	46 (50.0%)	38 (82.6%)	< 0.01
Myelodysplastic syndrome	46 (50.0%)	8 (17.4%)	< 0.01
ELN2022 adverse risk	37 (40.2%)	30 (65.2%)	0.69
IPSS‐R high or very high	27 (29.3%)	8 (17.4%)	0.07
Complex karyotype	20 (21.7%)	15 (32.6%)	0.25
*TP53* mutation	9 (9.8%)	9 (19.6%)	0.24
Chromatin modifier mutation (EZH2, ASXL1)	23 (25.0%)	13 (28.3%)	1.00
DNA methylation mutation (DNMT3A, IDH1, IDH2, TET2)	29 (31.5%)	17 (37.0%)	0.93
RAS pathway mutation (NRAS, KRAS, PTPN11, FLT3, KIT)	13 (14.1%)	15 (32.6%)	0.04
Tumor suppressor mutation (WT1, ETV6, PHF6)	9 (9.8%)	7 (15.2%)	0.64
Matched related donor	31 (33.7%)	10 (21.7%)	0.18
Matched unrelated donor	49 (53.3%)	33 (71.7%)	0.18
Mismatched unrelated donor	4 (4.3%)	0 (0%)	0.18
Haploidentical sibling	6 (6.5%)	3 (6.5%)	0.18
< 5% bone marrow blasts preSCT	72 (78.3%)	38 (82.6%)	0.60
Complete response at transplant	59 (64.1%)	33 (71.7%)	1.00
High or very high DRI	55 (59.8%)	29 (63.0%)	0.85
HCT‐CI ≥ 3	43 (46.7%)	25 (54.3%)	0.51
Nonmyeloablative or reduced intensity conditioning	58 (63.0%)	37 (80.4%)	0.06
Methotrexate GVHD prophylaxis	66 (71.7%)	22 (47.8%)	< 0.01
Posttransplant cyclophosphamide GVHD prophylaxis	19 (20.7%)	23 (50.0%)	< 0.01

**FIGURE 1:**
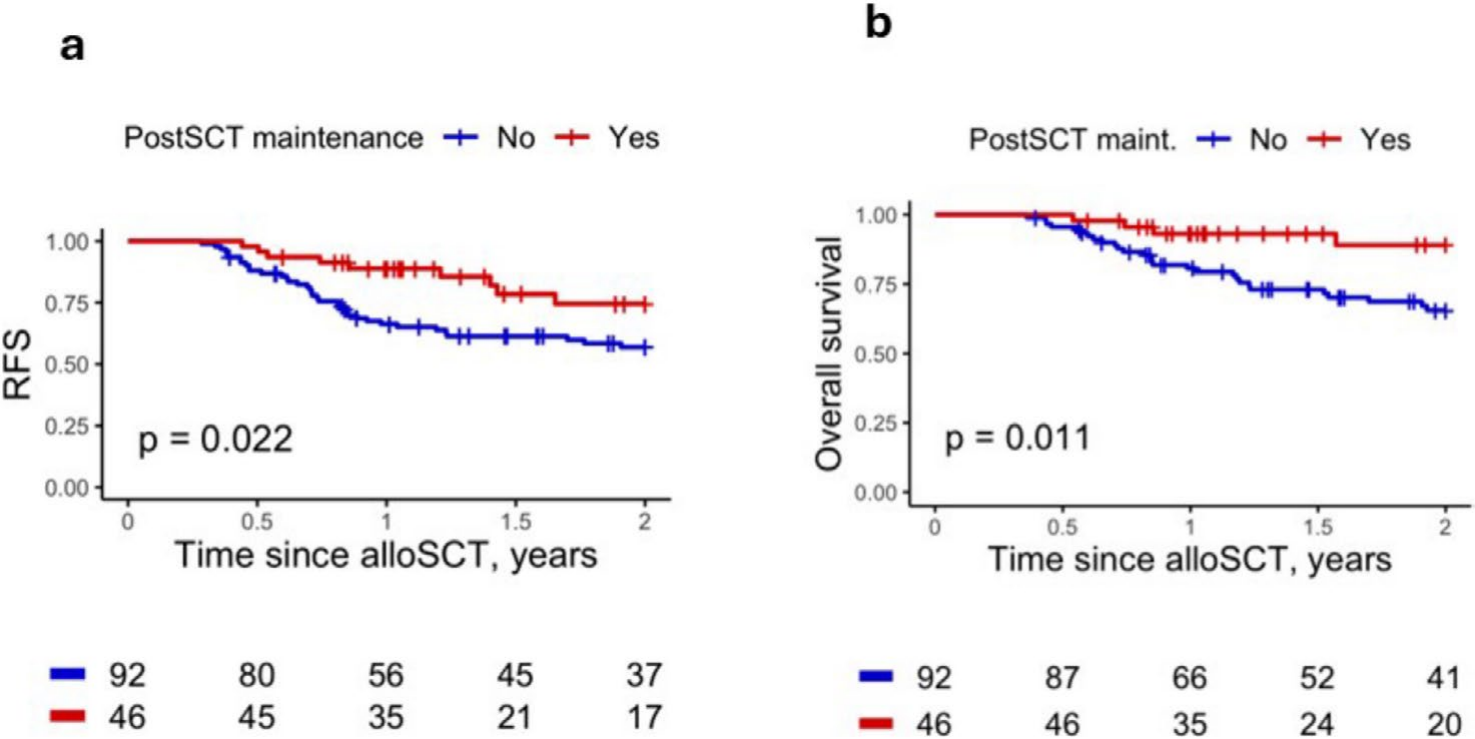
Kaplan–Meier curves of relapse free survival (RFS) (a) and overall survival (OS) (b) in propensity‐score matched low dose decitabine + rhG‐CSF maintenance (LD‐GD) vs. control cohorts.

Forty‐two patients (30.4%) received post‐transplant cyclophosphamide (PTCy) as GVHD prophylaxis; 23 (55.8%) of whom received LD‐GD maintenance. The median follow‐up of these patients was 12.8 months (95% CI: 11.3–18.5). In patients receiving PTCy, LD‐GD was associated with a significantly lower 1‐year cumulative incidence of relapse (8.7 vs. 40.7%, *p =* 0.049, Figure S8).

In multivariate competing risks analysis for relapse, LD‐GD was associated with a significantly decreased risk of relapse (HR 0.28, 95% CI: 0.0996–0.78, *p =* 0.015). Other prognostic factors included: complex karyotype (HR 3.72, 95% CI: 1.51–9.14, *p* = 0.004), CR at transplant (HR 0.33, 95% CI: 0.14–0.80, *p =* 0.014), and high/very high DRI (HR 4.45, 95% CI: 1.03–19.26, *p* = 0.046) (Table S4).

In multivariate overall survival analysis, LD‐GD maintenance was associated with a significantly improved 2‐year OS (HR 0.28, 95% CI 0.08–0.95, *p =* 0.04), while high/very high DRI was associated with worse 2‐year OS (HR 2.42, 95% CI: 1.03–5.72, *p =* 0.04) (Table S5).

Here, we report our experience using LD‐GD maintenance in HR‐MN following allo‐HSCT. This marks the first real‐world experience of LD‐GD maintenance therapy. Our findings build upon previous literature by including an older patient population receiving a reduced intensity conditioning regimen, including both AML and MDS. Overall, LD‐GD was feasible and well tolerated; patients received a median of 4 cycles. The grade 3–4 adverse event rate was 50%, primarily related to infection and cytopenias. Although adverse event rates were higher than reported in Gao et al., our rates of severe aGVHD and steroid‐requiring cGVHD were low at 10% and 22%, respectively. When compared to propensity‐matched control patients, patients with LD‐GD demonstrated significantly improved survival and decreased relapse rates. These findings remained consistent in patients receiving PTCy.

Our study is limited by the retrospective design, lacking data on intent to treat, especially for those who did not receive maintenance due to early relapse, provider decisions, or patient comorbidities. Although we attempted to compare similar patient populations and isolate the impact of LD‐GD maintenance using propensity matching on HCT‐CI and DRI, the retrospective design precludes drawing causal conclusions.

There was also a statistically significant difference between our maintenance and control cohorts' representation of primary diagnosis. The control cohort was enriched with MDS compared to the maintenance cohort (41% vs. 17.4%, *p =* 0.005). Due to limited control sample size, we were unable to propensity match to diagnosis and utilized DRI to counterbalance this discrepancy in diagnosis. MDS carries a worse prognosis compared to AML, with increased incidence of relapse [3] which was supported by the increased hazard ratio of MDS (HR 2.57, *p =* 0.009) in univariate analysis. Therefore, our results could be partially driven by the higher proportion of AML in the maintenance cohort compared to the control cohort.

Additional differences between cohorts included younger patients (61.4 vs. 65.9 years, *p =* 0.008), higher rates of myeloablative conditioning (37% vs. 19.6%), and decreased rates of RAS mutations (32.6% vs. 14%, *p =* 0.04) in the control cohort compared to the LD‐GD cohort. Previous literature has demonstrated that both age and *RAS* mutations negatively impact transplant outcomes [4]. Our data demonstrated that despite advanced age, the maintenance cohort had better outcomes.

GVHD prophylaxis regimens differed between the two groups, with significantly higher PTCy in the maintenance cohort compared to the control (50% vs. 20.7%, *p =* 0.0015). This may have contributed to the modest rates of GVHD in our maintenance cohort. However, in a dedicated subgroup analysis of patients who received PTCy, LD‐GD maintenance remained associated with improved outcomes.

The mechanism of LD‐GD is not fully understood, but decitabine has been shown to enhance both NK cell and effector T cell activity, which may act synergistically with the expansion of NK cell populations from rhG‐CSF [5] to improve the graft versus leukemia effect.

Recently, several targeted therapies have demonstrated improvements in relapse rates as post‐HSCT maintenance therapies [6], patients who lack targetable mutations may benefit from LD‐GD maintenance. Our findings would support the use of LD‐GD in these high‐risk patients, and further prospective randomized studies would be beneficial to further quantify the benefit of LD‐GD maintenance.

## Author Contributions

Mathias Palmer: data collection, cohort statistical analysis, initial manuscript drafting. Anmol Baranwal: data collection, matched pair analysis, figure generation, manuscript editing. Rami Basmaci and Khalil Hassan: data collection and manuscript review. Jade Kutzke, Gabriel Bartoo: manuscript review. Hemant Murthy, James Foran, Abhishek A. Mangaonkar, Mehrdad Hefazi, Aasiya Matin, William J. Hogan, Mark Litzow and Mithun Vinod Shah: contributed patients and manuscript review. Hassan B. Alkhateeb: study conceptualization, contributed patients, data collection, data analysis, manuscript drafting, editing, and review.

## Ethics Statement

There are no conflicts of interest to disclose. This study was approved by the Mayo Clinic Institutional Review Board.

## Conflicts of Interest

Some of the data was presented at the European Hematology Association 2024 conference.

## Supporting information


Data S1.

## Data Availability

The data that support the findings of this study are available from the corresponding author upon reasonable request.
