# Supplementary material for: Outcomes of Post‐Transplant Rh‐GCSF and Decitabine Maintenance Therapy in Patients With High‐Risk Myeloid Neoplasm
Source: Am J Hematol. 2025 Jul 29;100(10):1891–4. doi: 10.1002/ajh.70018 (PMC12417765; doi:10.1002/ajh.70018)
Supplement: Supplementary file 1 — Data S1. [file AJH-100-1891-s001.docx]

# Supplemental Information:

## Methods

### Patient Selection

This study was approved by the Mayo Clinic Institutional Review Board. All data was collected retrospectively from the electronic health record at the Mayo Clinic institutional sites located in Rochester, MN and Jacksonville, FL. Patients who received LD-GD following allo-HSCT for high-risk myelodysplastic syndrome (MDS), acute myeloid leukemia (AML), chronic myelomonocytic leukemia (CMML), or mixed-phenotype acute leukemia (MPAL) from 2020-2023 were identified. The control cohort included patients with high-risk MDS or AML who underwent allo-HSCT between 2018-2023 who did not receive maintenance therapy.

### Disease Stratification and Definitions

Risk stratification for AML and MDS was performed per ELN 2022 criteria and IPSS-R/IPSS-M, respectively. Molecular mutations were grouped into RAS pathway (*NRAS, KRAS, PTPN11, NF1*), DNA methylation (*DNMT3A, IDH1, IDH2, and TET2*), chromatin modifiers (*EZH2, ASXL1*), tumor suppressor genes (*WT1, ETV6, and PHF6*), RUNX1, and TP53 mutations. Disease risk index (DRI) was calculated based on results from Armand *et al*.

Conditioning regimen intensity was defined as per CIBMTR criteria.​ Acute GVHD was defined as per Glucksberg criteria and chronic GVHD was defined as per the 2014 National Institutes of Health (NIH) consensus criteria.​​ GVHD-free, relapse-free survival (GRFS) was defined as per the Blood & Marrow Transplant Clinical Trials Network Report.​

### Treatment

Patients in the LD-GD group received a maximum of 6 cycles of maintenance therapy (rhG-CSF was administered as 100 µg/m^2^ injection on days 0-5, and decitabine at 5 mg/m^2^ infusion on days 1-5 in accordance with Gao *et al*) every 6-8 weeks. Maintenance therapy was initiated provided the patient achieved engraftment with adequate count recovery in the absence of GVHD or relapse. Patients who received any other maintenance treatment after transplant to prevent relapse, such as sorafenib, gilteritinib, etc., were excluded. GVHD prophylaxis was assessed at day 100 and weaned based on individual provider discretion.

### Outcomes

Primary outcomes in the LD-GD analysis included median overall survival (mOS), relapse free survival (mRFS) and predicted OS and RFS at 1 and 2 years. Primary outcomes in the matched-pair analysis included time to disease relapse, non-relapse mortality, GVHD, and death.

Secondary outcomes included rates of adverse events, Grade III-IV aGVHD, and steroid-requiring cGVHD.

### Statistical Analysis

Patient and transplant characteristics were summarized using descriptive statistics. Categorical variables were analyzed using the chi-square test and continuous variables were analyzed using Kruskal-Wallis test for comparison of medians.

### Cohort Analysis:

LD-GD maintenance cohort mOS, mRFS, and estimated 1 and 2-year OS and RFS were assessed using Kaplan-Meier methodology. All statistical analysis was performed using BlueSky (V10.3.1).

### Matched Pair Analysis

Patients with AML or MDS in the LD-GD maintenance cohort were matched to a control cohort of consecutive patients with AML and MDS in a ratio of 1:2 using propensity score matching. Variables were considered balanced if the standardized mean difference (SMD) between the maintenance and control cohort was < 0.1. ​Patients were matched for a high HCT-CI score and high/very high DRI. Both were deemed categorical variables. Patients with relapse before day +100 were excluded from analysis.

The cumulative incidence of relapse was determined using competing risk analysis, with non-relapse mortality (NRM) considered as competing risk. Gray’s analysis was used to compare differences between cumulative incidence curves. Fine-Gray analysis was used for competing risk regression analyses. RFS and OS from transplant were determined using Kaplan-Meier and log-rank methods.​ Median follow-up time was determined using the reverse Kaplan-Meier method.​ Cox-proportional hazard method was used to evaluate factors impacting survival. Assumption of proportionality was verified for the Cox model. For purposes of survival analysis, “GVHD comorbidity” was defined as the development of grade III-IV acute GVHD or chronic GVHD requiring systemic therapy. Post-transplant maintenance and GVHD comorbidity were considered time-dependent covariates to assess their impact on RFS and OS.

Variables with *P*≤ 0.10 in univariate competing risk analysis (Table S7, Table S8) were included in the multivariate competing risk analysis for relapse. Factors included in multivariate model of relapse were LD-GD maintenance, chromatin modifiers, complex karyotype, CR at transplant and high/very high DRI. *TP53* mutation, monosomal karyotype, abnormal chromosome 17 were not included to prevent collinearity with complex karyotype.

Because a high number of variables had *P*< 0.1 in univariate analysis for RFS and OS, those variables were included in stepwise regression analysis with backward selection to arrive at the final multivariate model.

Factors included in stepwise reduction of 2-year OS were LD-GD maintenance, GVHD comorbidity, disease (AML vs MDS), TP53 mutation, RUNX1 mutation, DNA methylation mutation, CK, high/very high DRI, GVHD comorbidity and secondary AML/MDS.

All statistical analyses were performed using R 4.2.0 (R foundation for Statistical Computing, Vienna, Austria),​ R Core Team.​ Level of significance was set at *P*< 0.05.

## Results

### Maintenance Duration:

For patients who did not receive 6 cycles of LD-GD, 9 (18%) had a planned treatment of 4 cycles, 17 (34%) stopped maintenance early due to side effects, 3 (6%) relapsed while on maintenance, 5 (10%) were undergoing active treatment at the time of data collection, 1 (2%) stopped due to patient preference, and 1 (2%) pursued treatment at another medical center.

### LD-GD Patient Cohort Characteristics

The cytogenetic profile included 17 patients (34%) with CK, 4 (8%) monosomal karyotype (MK), and 12 (24%) CK+MK. Molecular analysis demonstrated 16 patients (32%) with RAS pathway, 18 (36%) with DNA methylation, 13 (26%) with chromatin modifier, and 6 (12%) with tumor suppressor mutations, while 9 (18%) had multihit *TP53* loss by ICC criteria.

At transplant, most AML patients were in complete response (CR) (25, 62.5%) or CRi (10, 25%). MRD by flow cytometry at the time of transplant was available in 33 (82%) patients and was positive in 8 (24%) of those patients. MRD status prior to maintenance therapy was available for 32 (80%) patients. Most patients with MDS were in marrow CR (6, 75%) and only 2 patients (25%) had active disease.

### LD-GD Molecular and Cytogenetic Subgroup Analysis

In subgroup analysis, median OS was 29.1 months (IQR: 29.1 - NA) for multihit *TP53* loss (Figure S9a) and 29.1 months (IQR:10.35 - NA) for complex monosomal karyotype (Figure S9b). The median RFS was 34.5 months (IQR: 34.5 – NA) for tumor suppressor mutations subgroup, 34.5 months (IQR: 34.5 – 34.5) for *RUNX1*, and 17.4 months (IQR: 6.57 – NA) for multihit *TP53* loss. Neither median OS nor RFS were reached in *RAS* Pathway, DNA methylation, nor chromatin modifier mutation subgroups (Table S2-3).

## Supplemental Figures

Figure S1: Kaplan Meier curves of LD-GD cohort for both median overall survival (OS) (a) and relapse free survival (RFS) (b).
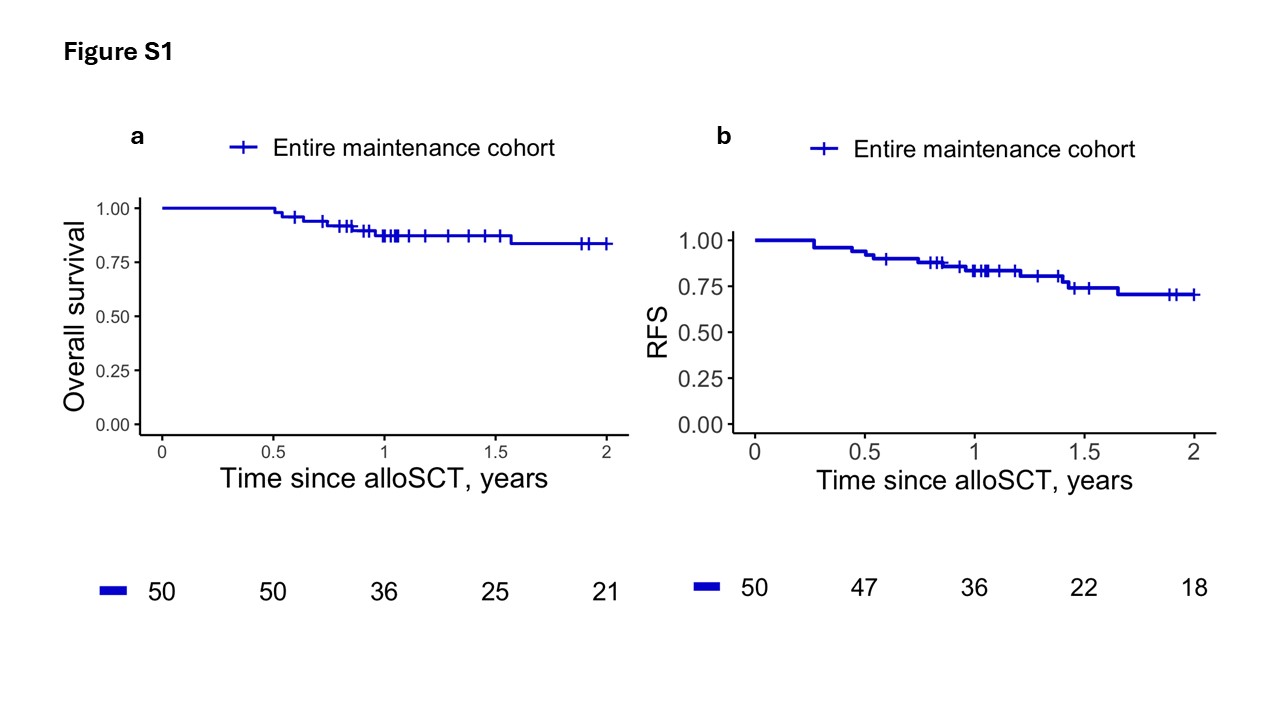


Figure S2: Competing risk analysis of LD-GD cohort demonstrating relapse and non-relapse mortality.


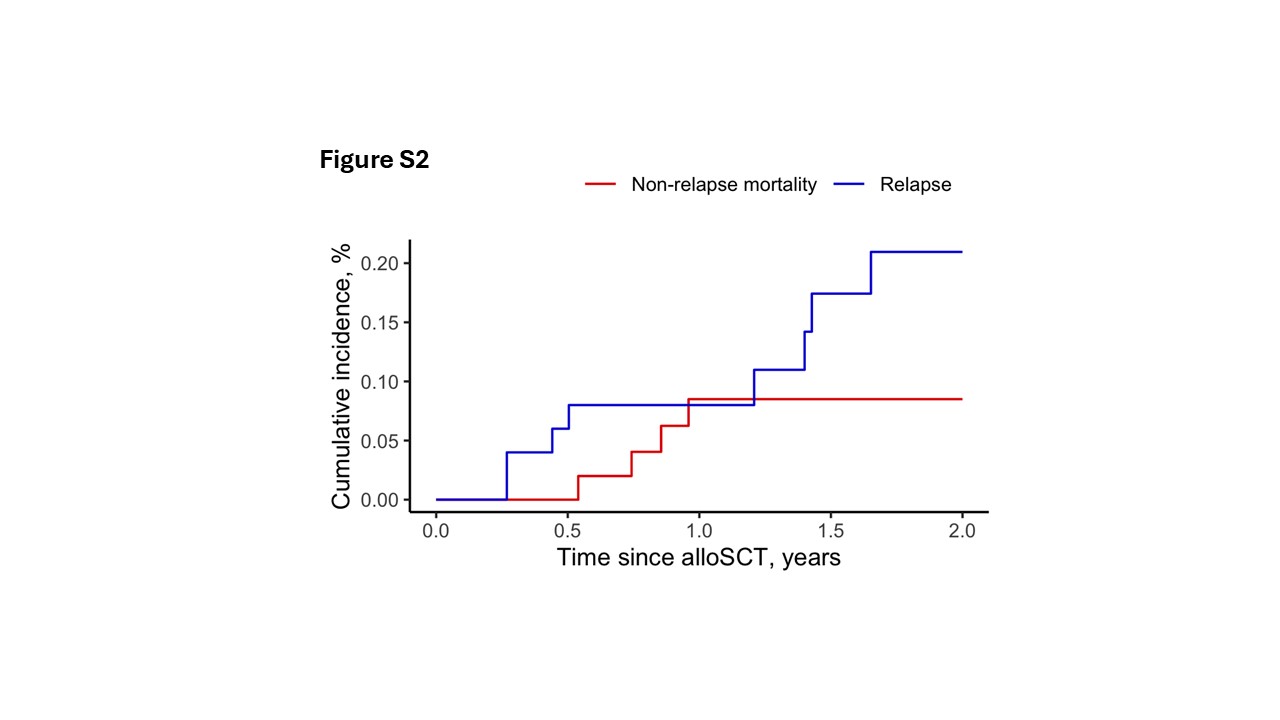


Figure S3: Mean difference reflecting covariate balancing using high HCT-CI and high or very high DRI in adjusted and unadjusted cohorts.


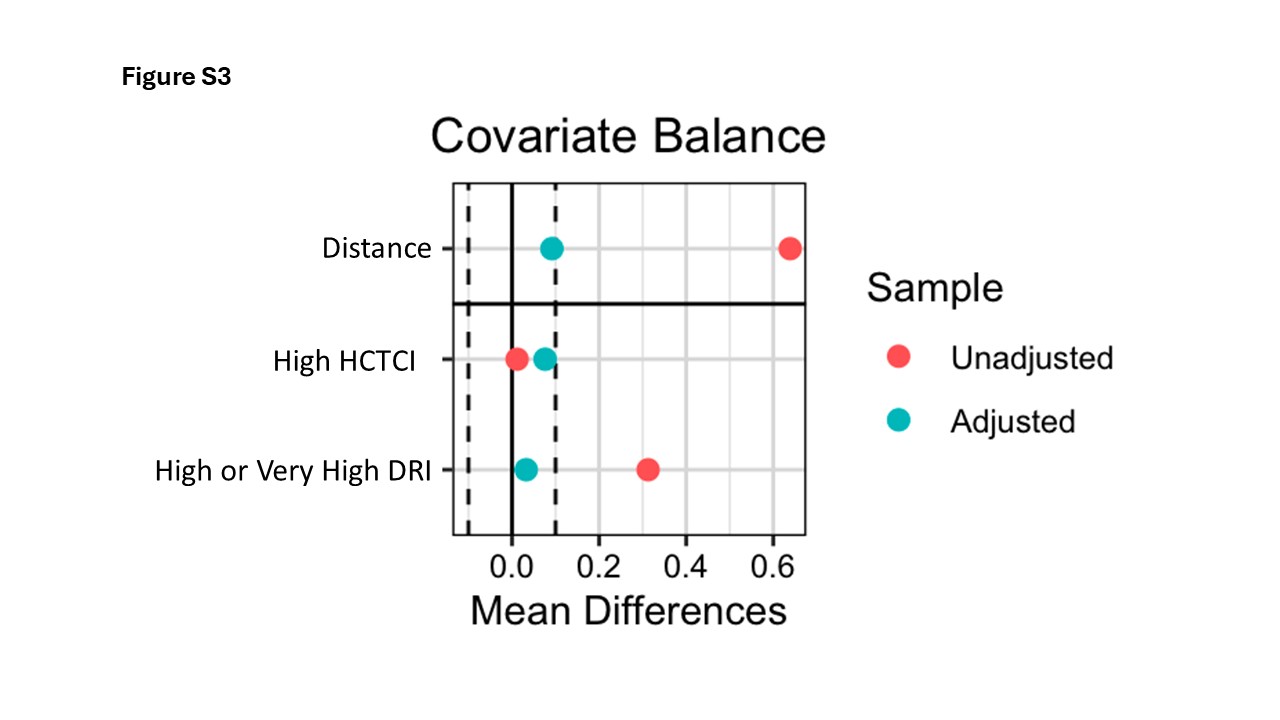


Figure S4: Distribution of patients with Pre-Transplant complete response (CR), relapse at 2 years, and myelodysplastic syndrome (MDS) (A) and acute myeloid leukemia (AML) (B) within the match pair cohort.


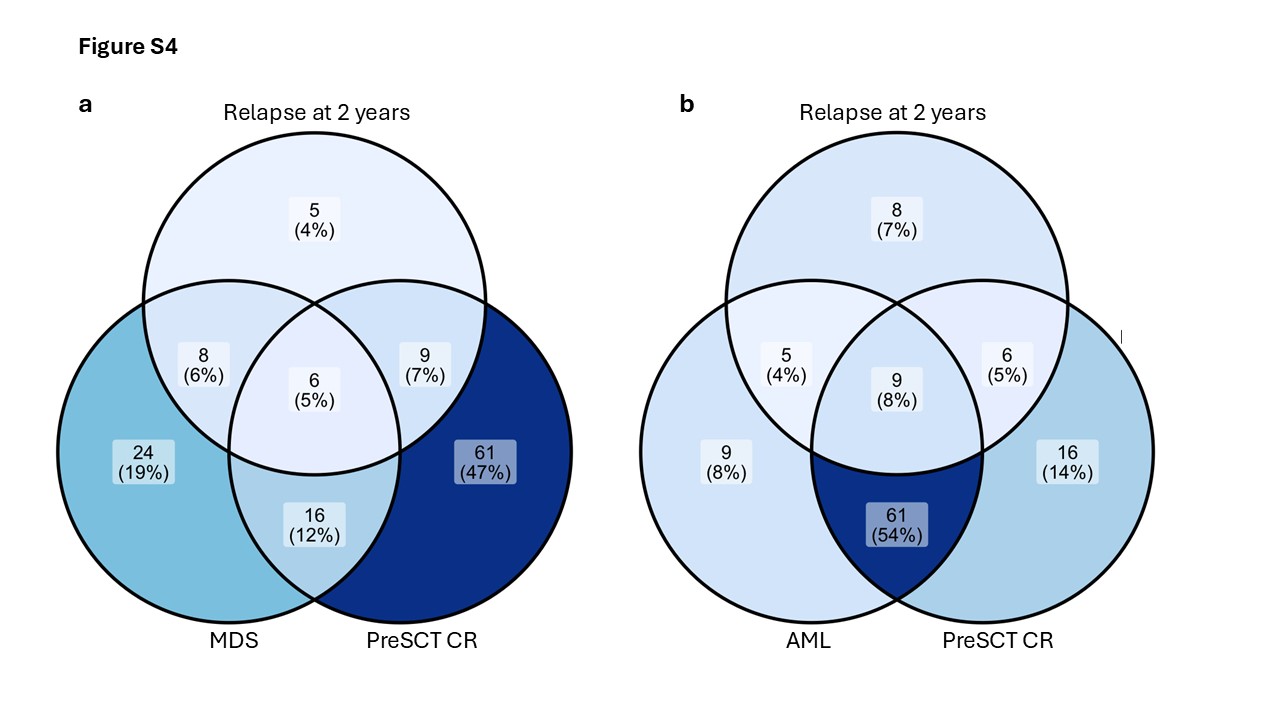


Figure S5: Cumulative incidence of non-relapse mortality and relapse in the propensity matched patient cohort with and without post allo-HSCT LD-GD therapy.


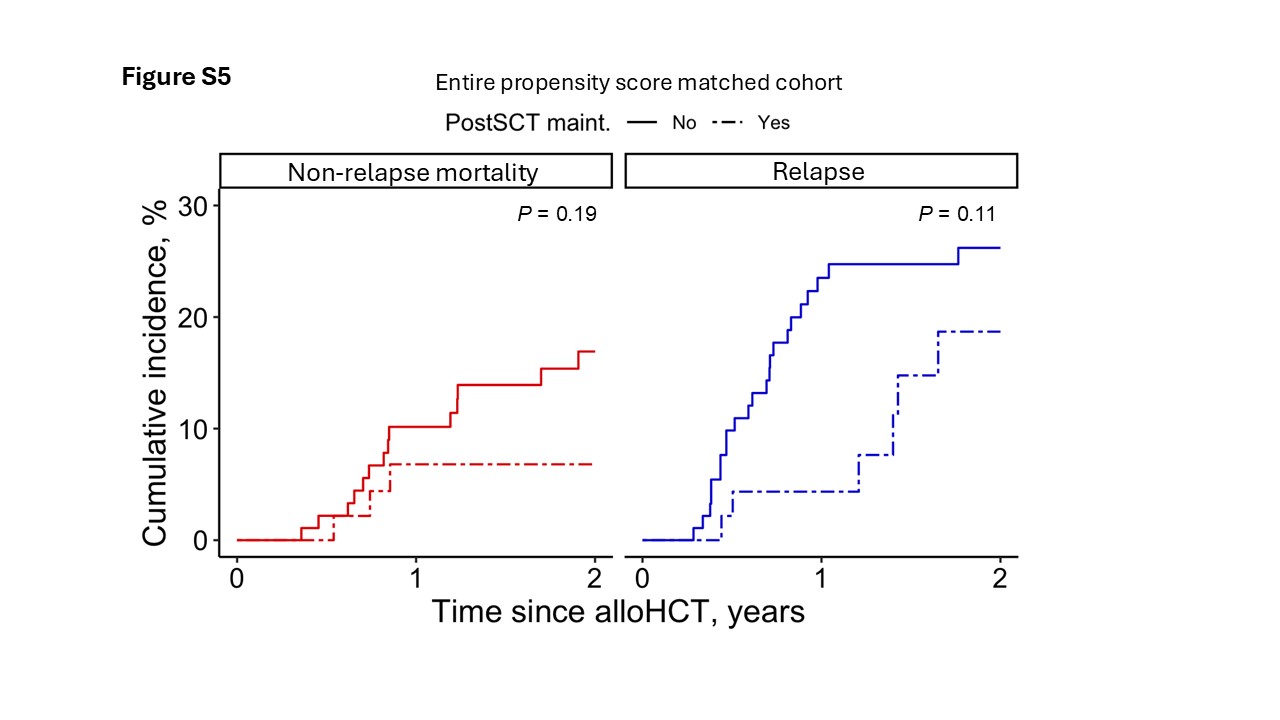


Figure S6: Relapse free survival (RFS) of propensity matched patient cohort of post allo-HSCT LD-GD without (a) and with (b) LD-GD as time-dependent variable.

#
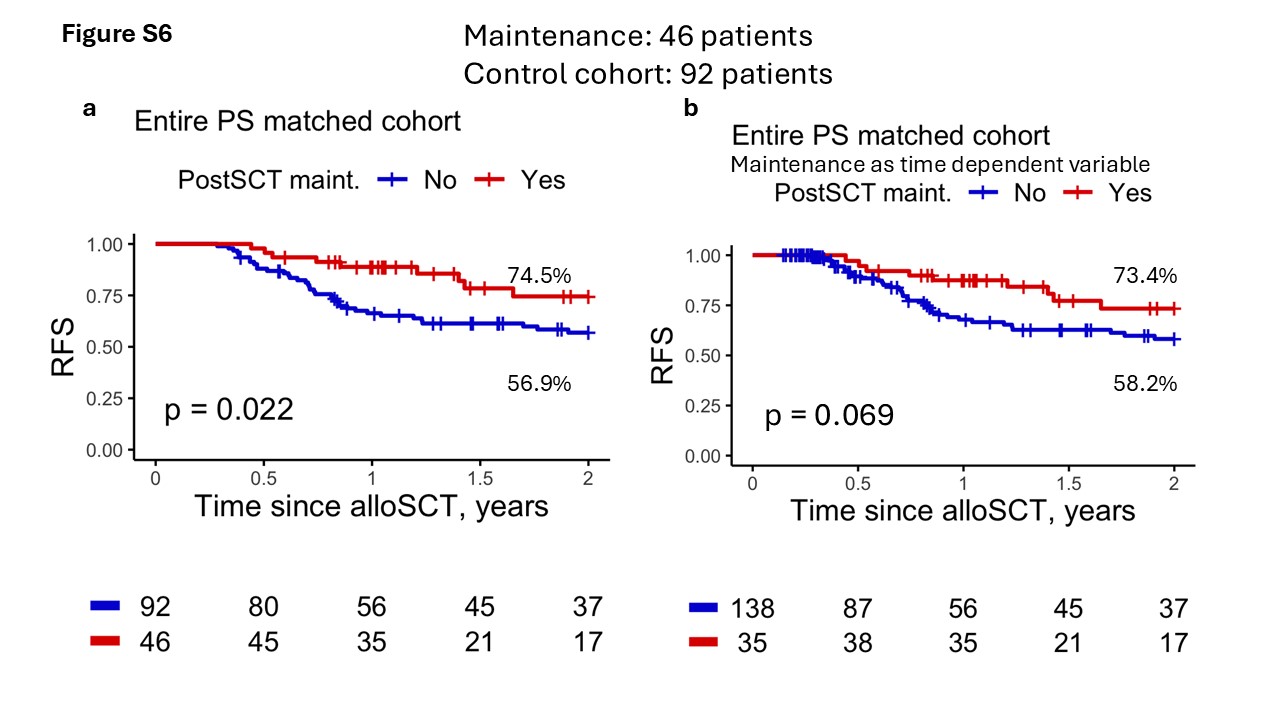


Figure S7: Overall survival of propensity matched patient cohort with of post allo-HSCT LD-GD without (a) and with (b) LD-GD as time-dependent variable.

#
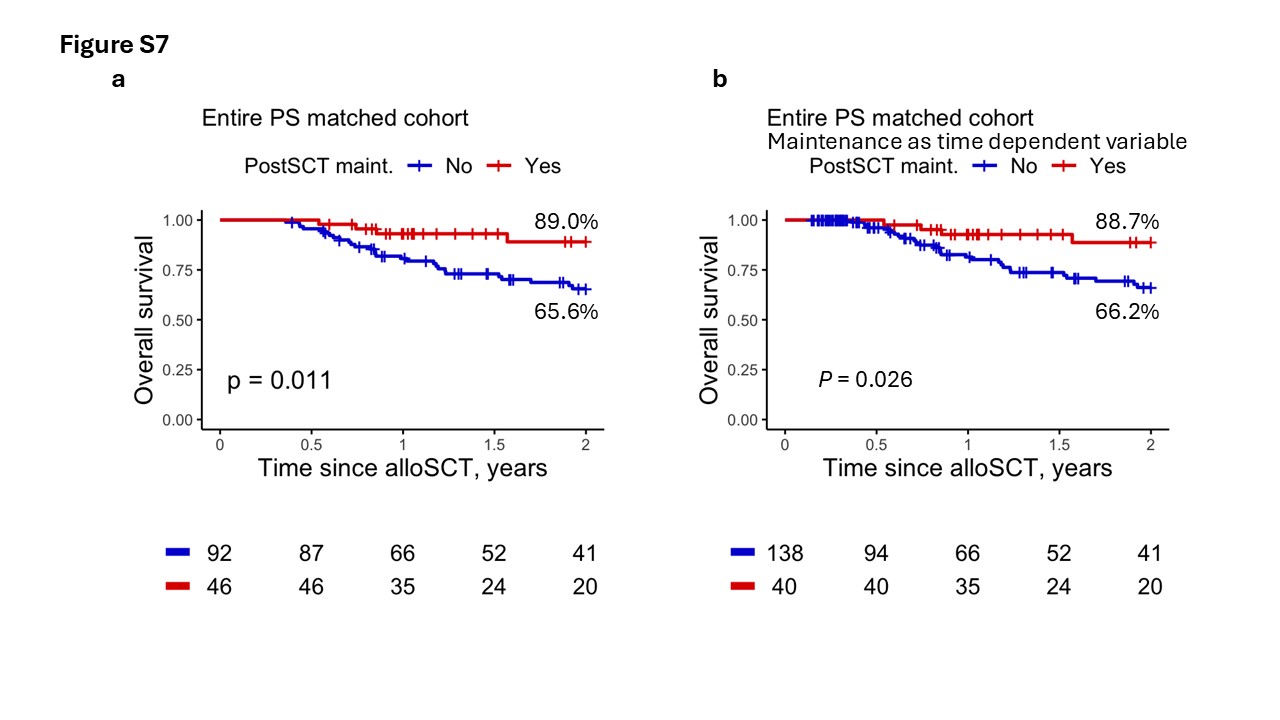


Figure S8: Comparison of non-relapse mortality and relapse rates in patients who received post-transplant cyclophosphamide (PTCy) according to post-transplant LD-GD.

#
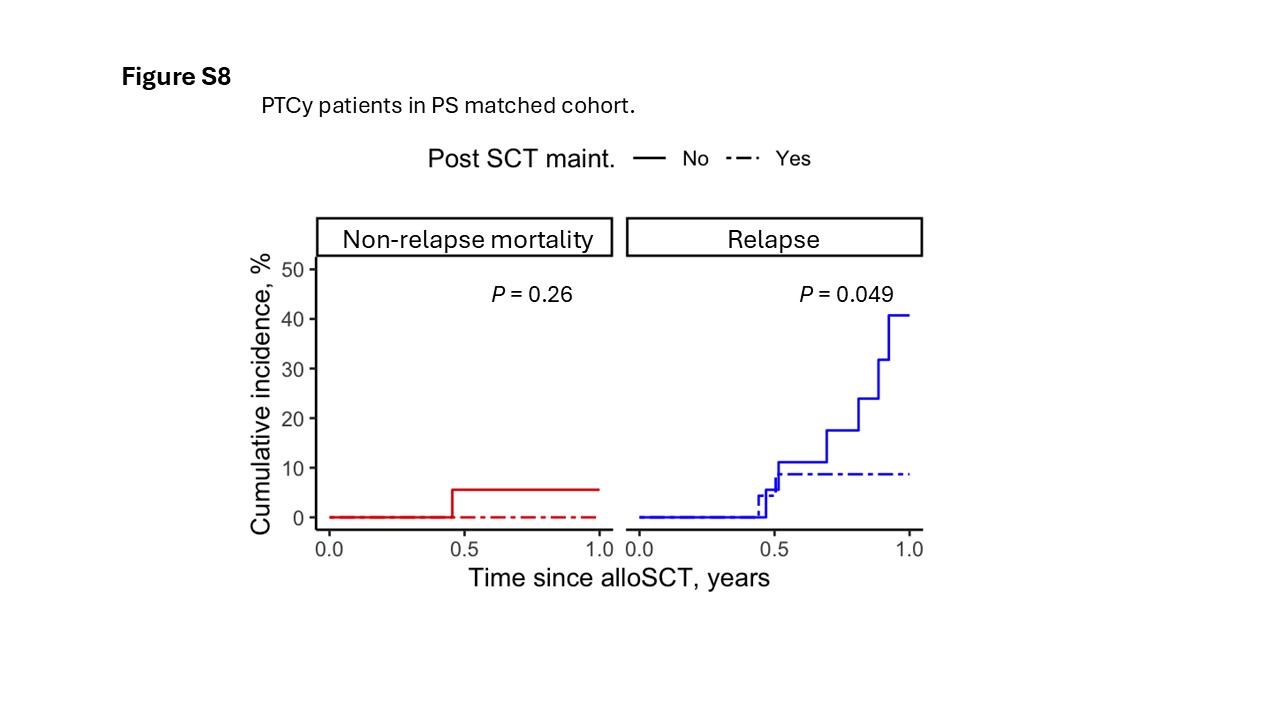


Figure S9: Kaplan Meier curves of multi-hit TP35 (a) and complex monosomal karyotype in patients who received LD-GD.


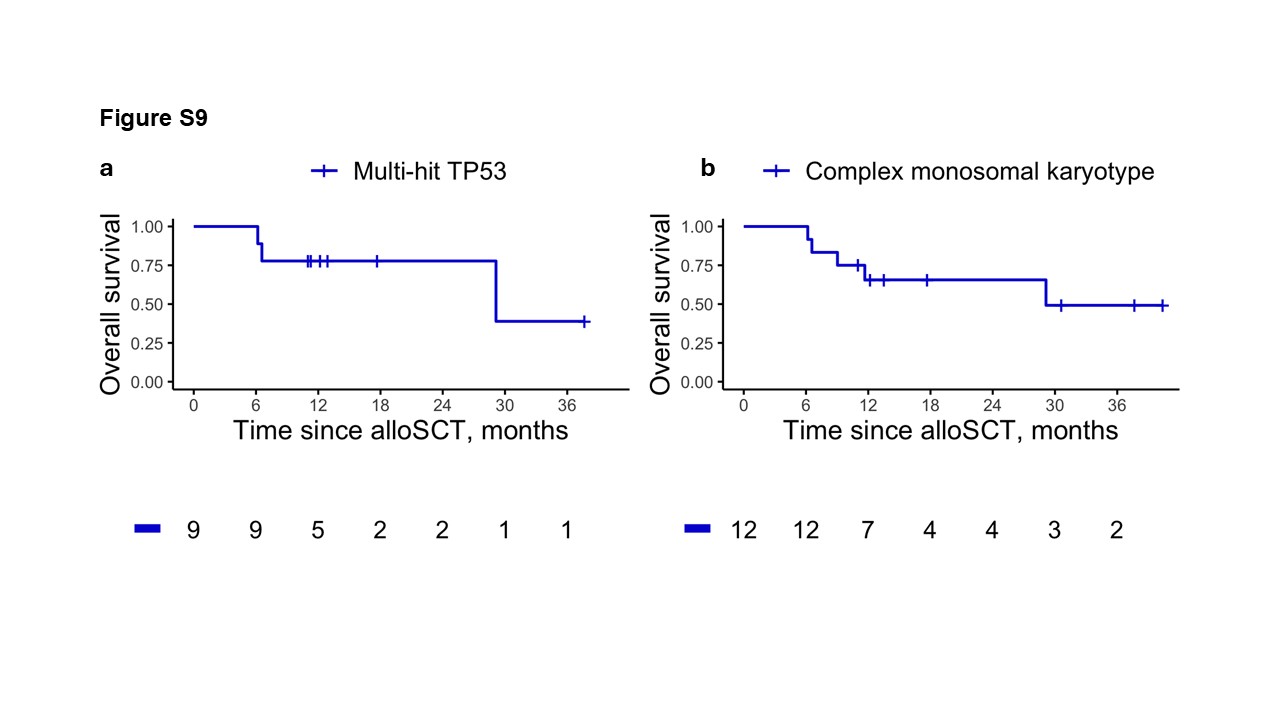


Supplemental Table 1: Patient characteristics of LD-GD Cohort

| Characteristic | LD-GD Cohort (n=50) |
| --- | --- |
| Sex (Female); n (%) | 25 (50) |
| Age at Transplant; median(range) | 65 (26-76) |
| Diagnosis |  |
| AML; n (%) | 40 (80) |
| MDS; n (%) | 8 (16) |
| MPAL; n (%) | 1 (2) |
| CMML; n (%) | 1 (2) |
| Karyotype |  |
| Complex Karyotype; n (%) | 17 (34) |
| Monosomal Karyotype; n (%) | 4 (8) |
| Complex + Monosomal Karyotype; n (%) | 12 (24) |
| Chromosome 17 abnormality; n (%) | 9 (18) |
| Chromosome 7 abnormality; n (%) | 15 (30) |
| AML ELN 2022 Classification |  |
| 1 - Favorable; n (%) | 2 (4) |
| 2 - Intermediate; n (%) | 7 (14) |
| 3 - Adverse; n (%) | 31 (62) |
| MDS IPSS-R |  |
| 1 - Very Low; n (%) | 0 |
| 2 - Low; n (%) | 0 |
| 3 - Intermediate; n (%) | 0 |
| 4 - High Risk; n (%) | 4 (50) |
| 5 - Very High Risk; n (%) | 4 (50) |
| MDS IPSS-M |  |
| 1 - Very Low; n (%) | 0 |
| 2 - Low; n (%) | 0 |
| 3 - Intermediate; n (%) | 0 |
| 4 - High Risk; n (%) | 2(25) |
| 5 - Very High Risk; n (%) | 6(75) |
| Genetic Mutations |  |
| TP53; n (%) | 10 (20) |
| TP53 Bi-Allelic; n (%) | 9 (18) |
| Mutation Class |  |
| RAS Pathway (NRAS, KRAS, PTPN11, CBL, NF1, RIT1, FLT3, KIT) 1=yes, 2=no; n (%) | 16 (32) |
| DNA Methylation (DNMT3A, IDH1, IDH2, TET2) Yes=1, no=0; n (%) | 18 (36) |
| Chromatin Modifiers (EZH2, ASXL1, KMT2A) 1=yes, 0=no; n (%) | 13 (26) |
| Tumor Suppressor Genes (TP53, WT1, ETV6, PHF6); n (%) | 17 (34) |
| Initial Treatment |  |
| Received HMA; n (%) | 32 (64) |
| HMA Resistant; n (%) | 3 (9.4) |
| Primary Refractory Disease; n (%) | 10 (20) |
| AML Response |  |
| CR1; n (%) | 28 (70) |
| CR2; n (%) | 4 (10) |
| CR3; n (%) | 2 (5) |
| Active Disease; n (%) | 6 (15) |
| Disease Status Prior to Transplant |  |
| CR; n (%) | 27 (54) |
| CRi; n (%) | 11 (22) |
| Active Disease; n (%) | 7 (14) |
| Marrow CR; n (%) | 5 (62.5) |
| Donor Type |  |
| MUD; n (%) | 36 (72) |
| MRD; n (%) | 11 (22) |
| Haploidentical; n (%) | 3 (6) |
| HLA Matching |  |
| 10/10; n (%) | 42 (84) |
| 9/10; n (%) | 5 (10) |
| Haploidentical; n (%) | 3 (6) |
| DP Matching |  |
| Matched; n (%) | 42 (84) |
| Permissive; n (%) | 5 (10) |
| Non-Permissive; n (%) | 0 (0) |
| MRD Positive at Transplant; n (%) | 8 (16) |
| MRD Positive at Maintenance; n (%) | 2 (4) |
| MRD Positive → negative; n (%) | 6 (75) |
| MRD negative → positive; n (%) | 0 (0) |
| Conditioning Regimen |  |
| Non-Myeloablative; n (%) | 2 (4) |
| Reduced Intensity Conditioning; n (%) | 40 (80) |
| Myeloablative Conditioning; n (%) | 8 (16) |
| Patient status at transplant |  |
| KPS < 80%; n (%) | 6 (12) |
| HCT-CI > 3; n (%) | 14 (28) |
| MRD Positive at Maintenance; n (%) | 2 (4) |
| Disease Status Prior to Maintenance |  |
| CR; n (%) | 43 (86) |
| CRI; n (%) | 3 (6) |
| Active Disease; n (%) | 0 (0) |
| Marrow Complete Response; n (%) | 4 (8) |
| Number of cycles; median (Range) | 4 (1-6) |
| Time From Transplant to Maintenance; median (IQR) | 3.6 months (1.4 - 9.9) |

Supplemental Table 2: Primary outcomes of median relapse free survival (RFS) and estimated RFS at times 1 and 2 years post-transplant.

| Cohort (n) | RFS (95% CI) | Est 1-yr RFS (95% CI) | Est 2-yr RFS (95% CI) |
| --- | --- | --- | --- |
| All Patients (50) | NR | 84 (74-95) | 71 (57-87) |
| Monosomal Karyotype (MK) (4) | NR | 75 (43-100) | 75 (43-100) |
| Complex Karyotype (CK) (17) | 17.4 (14.7-NA) | 70 (50-96) | 50 (28-88) |
| MK + CK (12) | 17.4 (9-NA) | 58 (36-0.94) | 47 (24-90) |
| RUNX1 (8) | 34.5 (NA-NA) | 88 (67-100) | 88 (67-100) |
| TP53 (10) | 17.4 (6.6-NA) | 70 (47-100) | 47 (19-100) |
| Biallelic TP53 (9) | 17.4 (6.6-NA) | 67 (42-100) | 44 (18-100) |
| RAS Pathway (NRAS, KRAS, PTPN11, FLT3, KIT) (16) | NR | 81 (64-100) | 61 (38-100) |
| DNA Methylation (DNMT3A, IDH1, IDH2, TET2) (18) | NR | 89 (75-100) | 79 (59-100) |
| Chromatin Modifiers (EZH2, ASXL1) (13) | NR | 92 (79-100) | 92 (79-100) |
| Tumor Suppressor (WT1, ETV6, PHF6) (17) | 34.5 (34.5-NA) | 100 (100-100) | 100 (100-100) |

Supplemental Table 3: Primary outcomes of median overall survival (OS) and estimated OS at times 1 and 2 years post-transplant.

| Cohort (n) | OS (95%CI) | Est 1-yr OS (95% CI) | Est 2-yr OS (95% CI) |
| --- | --- | --- | --- |
| All Patients (50) | NR | 87 (78-97) | 84 (73-96) |
| Monosomal Karyotype (MK) (4) | NR | 75 (43-100) | 75 (43-100) |
| Complex Karyotype (CK) (17) | NR | 75 (56-100) | 75 (56-100) |
| MK + CK (12) | 29.1 (11.7-NA) | 66 (43-100) | 66 (43-100) |
| RUNX1 (8) | NR | 100 (100-100) | 100 (100-100) |
| TP53 (10) | 29.1 (29.1-NA) | 80 (59-100) | 80 (59-100) |
| Biallelic TP53 (9) | 29.1 (29.1-NA) | 78 (55-100) | 78 (55-100) |
| RAS Pathway (NRAS, KRAS, PTPN11, FLT3, KIT) (16) | NR | 94 (83-100) | 94 (0.83-100) |
| DNA Methylation (DNMT3A, IDH1, IDH2, TET2) (18) | NR | 94 (0.83-100) | 94 (83-100) |
| Chromatin Modifiers (EZH2, ASXL1) (13) | NR | 100 (100-100) | 100 (100-100) |
| Tumor Suppressor (WT1, ETV6, PHF6) (17) | NR | 100 (100-100) | 100 (100-100) |

Supplemental Table 4: Multivariate regression competing risk analysis for 2 year relapse.

|  | Multivariate competing risk analysis for 2 year relapse | |
| --- | --- | --- |
| Variable | HR (CI) | P |
| LD-GD Therapy | 0.28 (0.0996 – 0.78) | 0.015 |
| Chromatin Modifiers (EZH2, ASXL1) | 0.39 (0.10 - 1.48) | 0.17 |
| Complex Karyotype | 3.72 (1.51 - 9.14) | 0.004 |
| Complete Response at Transplant | 0.33 (0.14 - 0.80) | 0.014 |
| High or Very High DRI | 4.45 (1.03 – 19.26) | 0.046 |

Supplemental Table 5: Multivariate regression cox proportional hazard analysis of 2 year overall survival (OS)

| \|  \| Multivariate regression cox proportional hazard analysis of 2 year OS \| \| \| --- \| --- \| --- \| \| Variable \| HR (CI) \| P \| \| LD-GD Therapy \| 0.29 (0.08 - 0.95) \| 0.04 \| \| RUNX1 \| 0.16 (0.02 - 1.21) \| 0.08 \| \| Secondary AML or MDS \| 0.21 (0.03 - 1.55) \| 0.12 \| \| High or Very High DRI \| 2.42 (1.03 - 5.72) \| 0.04 \| |
| --- | --- | --- | --- | --- | --- | --- | --- | --- | --- | --- | --- | --- | --- | --- | --- | --- | --- | --- |

Supplemental Table 6: Patient characteristics of LD-GD cohort and control cohort, excluding relapse before day + 100 and CMML/APML.

| Variable | Control (N=173) | LD-GD (N=46) | P |
| --- | --- | --- | --- |
| Median age at diagnosis [Min, Max] | 61.2 [18.9, 75.2] | 65.5 [26.2, 75.9] | < 0.01 |
| Median age at transplant [Min, Max] | 62.7 [19.1, 76.2] | 65.9 [26.6, 76.5] | < 0.01 |
| Sex (Female) | 62 (35.8%) | 22 (47.8%) | 0.19 |
| Acute myeloid leukemia | 102 (59.0%) | 38 (82.6%) | 0.01 |
| Myelodysplastic syndrome | 71 (41.0%) | 8 (17.4%) | 0.01 |
| ELN2022 adverse risk | 61 (35.3%) | 30 (65.2%) | 0.10 |
| IPSS-R high or very high | 39 (22.5%) | 8 (17.4%) | 0.04 |
| Complex karyotype | 25 (14.5%) | 15 (32.6%) | 0.01 |
| *TP53* mutation | 12 (6.9%) | 9 (19.6%) | 0.04 |
| Chromatin modifier mutation (EZH2, ASXL1) | 38 (22.0%) | 13 (28.3%) | 0.77 |
| DNA methylation mutation (DNMT3A, IDH1, IDH2, TET2) | 54 (31.2%) | 17 (37.0%) | 0.95 |
| RAS pathway mutation (NRAS, KRAS, PTPN11, FLT3, KIT) | 32 (18.5%) | 15 (32.6%) | 0.14 |
| Tumor suppressor mutation (WT1, ETV6, PHF6) | 17 (9.8%) | 7 (15.2%) | 0.61 |
| Matched Related Donor | 64 (37.0%) | 10 (21.7%) | 0.08 |
| Mismatched Unrelated Donor | 6 (3.5%) | 0 (0%) | 0.08 |
| Matched Unrelated Donor | 86 (49.7%) | 33 (71.7%) | 0.08 |
| Haploidentical Sibling | 13 (7.5%) | 3 (6.5%) |  |
| <5% Bone marrow blasts preSCT | 148 (85.5%) | 38 (82.6%) | 0.92 |
| Complete response at transplant | 131 (75.7%) | 33 (71.7%) | 0.17 |
| High or very high DRI | 55 (31.8%) | 29 (63.0%) | <0.01 |
| HCT-CI ≥ 3 | 92 (53.2%) | 25 (54.3%) | 1.00 |
| Non-myeloablative or reduced intensity conditioning | 112 (64.7%) | 37 (80.4%) | 0.06 |
| Methotrexate GVHD prophylaxis | 125 (72.3%) | 22 (47.8%) | < 0.01 |
| Post-transplant Cyclophosphamide GVHD prophylaxis | 40 (23.1%) | 23 (50.0%) | < 0.01 |

Supplemental Table 7: Univariate analysis of relapse free survival (RFS) in propensity matched cohort.

| Univariate Cox prop. hazard analysis for 2 year RFS | | |
| --- | --- | --- |
| **Variable** | **HR** | **P** |
| Age | 1.00 (0.98 - 1.03) | 0.96 |
| MDS (vs. AML) | 2.20 (1.22 - 3.96) | 0.01 |
| Maintenance Therapy (Time Dependent) | 0.45 (0.21 - 0.97) | 0.04 |
| ASXL1 | 0.43 (0.18 - 1.03) | 0.06 |
| TP53 | 3.03 (1.51 - 6.08) | < 0.01 |
| RUNX1 | 0.31 (0.10 - 0.99) | 0.05 |
| Tumor Suppressor (WT1, ETV6, PHF6) | 0.59 (0.21 - 1.66) | 0.32 |
| Chromatin Modifiers (EZH2, ASXL1) | 0.37 (0.16 - 0.88) | 0.02 |
| DNA Methylation (DNMT3A, IDH1, IDH2, TET2) | 0.50 (0.24 - 1.01) | 0.05 |
| RAS Pathway (NRAS, KRAS, PTPN11, FLT3, KIT) | 0.89 (0.41 - 1.93) | 0.77 |
| Monosomal Karyotype | 0.71 (0.17 - 2.92) | 0.63 |
| Complex Karyotype | 2.66 (1.47 - 4.81) | < 0.01 |
| Chromosome 17 Mutation | 2.95 (1.46 - 5.96) | 0.00 |
| Chromosome 7 Mutation | 2.01 (1.09 - 3.70) | 0.03 |
| Secondary AML or MDS | 0.58 (0.24 - 1.37) | 0.21 |
| Complete Response at Maintenance | 0.47 (0.26 - 0.86) | 0.02 |
| MRD Positive Prior to Transplant | 3.52 (1.18 - 10.49) | 0.02 |
| High or Very High DRI | 4.03 (1.87 - 8.68) | < 0.01 |
| High HCT-CI | 1.07 (0.60 - 1.92) | 0.82 |
| Myeloablative Conditioning | 0.97 (0.52 - 1.83) | 0.93 |
| PTCy | 1.08 (0.55 - 2.12) | 0.82 |
| Age > 65 | 1.17 (0.65 - 2.10) | 0.60 |
| Treatment after 2021 | 0.98 (0.54 - 1.78) | 0.95 |
| Total Body Irradiation | 0.81 (0.20 - 3.35) | 0.77 |
| Cytoxan | 1.11 (0.49 - 2.48) | 0.81 |
| Busulfan | 0.77 (0.40 - 1.47) | 0.43 |
| Melphalan | 1.02 (0.55 - 1.86) | 0.96 |
| WT1 | 0.37 (0.05 - 2.70) | 0.33 |
| BCOR | 0.84 (0.26 - 2.71) | 0.77 |
| GVHD comorbidity (as time dependent variable) | 1.69 (0.88 - 3.24) | 0.12 |

Supplemental Table 8: Univariate analysis of overall survival (OS) in propensity matched cohort.

| Univariate Cox prop. hazard analysis for 2 year OS | | |
| --- | --- | --- |
| **Variable** | **HR** | **P** |
| Age | 1.01 (0.98 - 1.04) | 0.73 |
| MDS (vs. AML) | 2.57 (1.27 - 5.21) | 0.01 |
| Maintenance Therapy (Time Dependent) | 0.30 (0.11 - 0.87) | 0.03 |
| ASXL1 | 0.58 (0.22 - 1.52) | 0.27 |
| TP53 | 2.32 (0.94 - 5.73) | 0.07 |
| RUNX1 | 0.15 (0.02 - 1.08) | 0.06 |
| Tumor Suppressor (WT1, ETV6, PHF6) | 0.92 (0.32 - 2.64) | 0.88 |
| Chromatin Modifiers (EZH2, ASXL1) | 0.49 (0.19 - 2.18) | 0.15 |
| DNA Methylation (DNMT3A, IDH1, IDH2, TET2) | 0.41 (0.17 - 1.01) | 0.05 |
| RAS Pathway (NRAS, KRAS, PTPN11, FLT3, KIT) | 0.57 (0.20 - 1.64) | 0.30 |
| Monosomal Karyotype | 0.49 (0.07 - 3.62) | 0.49 |
| Complex Karyotype | 1.99 (0.97 - 4.08) | 0.06 |
| Chromosome 17 Mutation | 2.10 (0.87 - 5.11) | 0.10 |
| Chromosome 7 Mutation | 1.81 (0.87 - 3.77) | 0.11 |
| Secondary AML or MDS | 0.24 (0.06 - 1.01) | 0.05 |
| Complete Response at Maintenance | 0.65 (0.31 - 1.35) | 0.24 |
| MRD Positive Prior to Transplant | 2.33 (0.56 - 9.78) | 0.25 |
| High or Very High DRI | 3.02 (1.30 - 7.01) | 0.01 |
| High HCT-CI | 2.26 (1.08 - 4.76) | 0.03 |
| Myeloablative Conditioning | 1.35 (0.67 - 2.71) | 0.40 |
| PTCy | 0.88 (0.42 - 1.86) | 0.74 |
| Age > 65 | 0.61 (0.23 - 1.59) | 0.31 |
| Treatment after 2021 | 1.14 (0.57 - 2.28) | 0.72 |
| Total Body Irradiation | 0.91 (0.45 - 1.83) | 0.79 |
| Cytoxan | 1.09 (0.53 - 2.25) | 0.81 |
| Busulfan | 0.79 (0.28 - 2.25) | 0.66 |
| Melphalan | 0.70 (0.32 - 1.52) | 0.37 |
| WT1 | 1.09 (0.53 - 2.25) | 0.81 |
| BCOR | 0.59 (0.08 - 4.35) | 0.61 |
| GVHD comorbidity (as time dependent variable) | 1.36 (0.41 - 4.49) | 0.62 |
